# Diagnosis and treatment of coronary artery disease in hemodialysis patients evaluated for transplant

**DOI:** 10.1186/2047-1440-1-3

**Published:** 2012-04-24

**Authors:** Jose JG De Lima, Luis Henrique W Gowdak, Flavio J de Paula

**Affiliations:** 1 Heart Institute (InCor), Hospital das Clínicas, University of São Paulo Medical School, Rua Eneas Carvalho Aguiar, 44, 05403-000,, São Paulo, Brazil; 2 Renal Transplant Unit, Urology, Hospital das Clínicas, University of São Paulo Medical School, Rua Eneas Carvalho Aguiar, 39, 05403-000, São Paulo, Brazil

**Keywords:** Chronic kidney disease, Coronary artery disease, Renal transplantation, Myocardial scintigraphy, Coronary angiography

## Abstract

We present a review of current strategies for the diagnosis and treatment of coronary artery disease (CAD) in patients with advanced chronic kidney disease who are on the waiting list for transplants, based on data from the literature and originated from a single-center cohort of 1,250 patients with maximum follow-up of 12 years. We discuss the best way to select patients to be tested for CAD, how to choose the more adequate screening test for CAD and cardiovascular disease, how to select patients for invasive treatment studies and how to treat patients with significant CAD. We also suggest new research avenues to be explored to resolve some problems in this area.

## Introduction

Compared to the general population, patients with chronic kidney disease (CKD) are at the highest risk of developing cardiovascular complications and dying [1]. This trend has been observed in individuals with moderate reduction in renal function and increases as renal insufficiency progresses [2]. The adjusted risk of cardiovascular death for patients on dialysis is 10 to 20 times higher than that in the general population, and 50% of such deaths are related to coronary artery disease (CAD) [3,4]. Also, patients with CKD have a worse prognosis once one event has occurred [5], thus most patients with CKD are more likely to die as a result of cardiovascular disease (CVD) than to reach the final stages of renal failure and be started on renal replacement therapy [6]. The prevalence of significant CAD (>50% stenosis) in dialysis patients varies between 30% and 70% and is greatly influenced by age and the presence of diabetes as well as by the use of angiography as a diagnostic method [7]‐[12]. Renal transplantation is associated with improved survival [13], but CVD remains the most common cause of death after transplantation [14]. Together, these facts justify the routine assessment of patients with CKD for associated CVD and CAD, including those being considered for kidney transplantation.

The optimal way to screen for and manage CAD prior to and following kidney transplantation is a topic of intense debate in the literature. There is no firm consensus about who should be tested, which testing modality should be used and who should undergo intervention if CAD is found [15]‐[15-18]. In 1997, our center started a prospective, observational study intended to determine the best clinical and cardiovascular investigations for the detection of CAD and the prediction of cardiovascular events in patients evaluated for kidney transplants. The cohort now encompasses 1,250 patients with a median follow-up of 38 months. The present article is based on the database and data collected during the follow-up periods, as well as on pertinent observations reported in the literature.

### Which patients should be tested for CAD?

According to the American Society of Transplantation (AST) guidelines, the elderly, patients with diabetes and patients with associated clinical CVD (high-risk patients) should be tested for CAD [15]. High-risk patients are clinically defined by clinical evidence of actual or past vascular arterial disease, heart failure and previous stroke or myocardial infarction. Which patients should be considered at low risk is unclear, however; that is, young patients (< 50 years old), patients without diabetes and those with no clinical evidence of CVD should also be tested, because CKD is considered an independent risk factor for cardiovascular death. Therefore, we tested the hypothesis that clinical stratification alone would make detailed cardiac evaluation unnecessary in low-risk patients [19]. We evaluated 363 consecutive candidates for renal transplantation followed up for 51 months. We found that only 1 of 93 low-risk patients had an adverse event (stroke), and no coronary events were observed. Contrary to that finding, 49 adverse events occurred in 270 high-risk patients, comprising 12 strokes, 12 sudden deaths, 11 cases of unstable angina, 6 myocardial infarctions, 4 congestive heart failures requiring hospitalization and 4 acute peripheral vascular events resulting in intervention. Of these adverse events, 29 (58%) were attributed to CAD (sudden cardiac death, myocardial infarction and unstable angina). These results indicate that in-depth cardiac investigation is not required in low-risk asymptomatic patients and that clinical stratification is an adequate tool to identify subjects at high risk for future cardiovascular events.

### What is the best screening test for CAD and cardiovascular events in high-risk patients?

Guidelines for the detection of CAD and assessment of risk in CKD patients are based on the results of noninvasive testing, such as myocardial scanning (dipyridamole stress testing single-photon emission-computed tomography (SPECT)) and echocardiography stress testing (EST) with dobutamine-atropine on the basis of data derived from the nonuremic population [20]. In the majority of cases, the accuracy of these tests in patients with CKD was evaluated by the incidence of adverse events, with the invasive test reserved for those patients with evidence of ischemia. On the basis of using this approach, the sensibility and specificity of SPECT have been found to be highly variable by diverse authors, usually lower than 80% [21]‐[26].

It is important to point out that epidemiological studies have indicated that a useful diagnostic test for a condition that is highly prevalent, such as CAD in CKD patients, must have sensitivity and specificity of at least 80%. The negative predictive value, which incorporates the disease prevalence in its formula, should be even higher. Otherwise, a negative test is likely to represent a false-negative result.

We evaluated prospectively the accuracy of these two noninvasive tests in detecting CAD (≥70% stenosis) and assessing cardiovascular risk using coronary angiography as the “gold standard” in 126 high-risk patients classified according to the AST criteria [8]. The prevalence of CAD was 42%. The sensitivity and negative predictive values of both noninvasive tests for the detection of CAD were <75%, meaning that the tests failed to identify almost one-third of patients with significant coronary stenosis. More importantly, CAD, but not the noninvasive tests, correlated with adverse events with a sensitivity of 86% and a negative predictive value of 96%. The relative risk of adverse events for patients with CAD was almost 10 times higher, and CAD was the only variable significantly related to adverse events in multivariate analysis. Therefore, we concluded that the current noninvasive tests are of limited usefulness to select high-risk patients for coronary angiography and for risk stratification.

Are there alternatives to SPECT and EST? Exercise electrocardiography may be helpful but cannot be used in many CKD patients because of their low tolerance to exercise [27]. Cardiac computed tomographic angiography has not been evaluated extensively in this population, and cardiac magnetic resonance imaging is no longer indicated for patients with renal disease, owing to the risk of gadolinium-associated systemic fibrosis [28]. In our center, the accuracy of the coronary calcium score as a predictor of CAD was found to be comparable to SPECT (area under the receiver operating characteristic curve = 0.70) [29]. We may say that there is still no totally satisfactory screening method for CAD in this group of patients.

### Should all high-risk patients undergo coronary angiography?

Because of the suboptimal performance of current noninvasive testing to detect CAD and stratify patients for cardiovascular risk, clinicians at some centers advocate the use of an invasive test for all high-risk patients. Certainly, relying only on noninvasive tests would cause 20% to 30% of high-risk patients with CAD to be undiagnosed. However, coronary angiography is expensive and not without risks. Moreover, the majority of studies have conclusively shown that the prevalence of CAD in the high-risk patients evaluated by angiography is, on average, close to 50%. This means that, if we accept that approach, a significant proportion of patients will be exposed to invasive testing with no clear clinical advantage. Therefore, in our center, we are working to find ways to identify, among high-risk patients, those with a higher probability of occult significant coronary stenosis and, as a consequence, more likely to benefit from angiography.

Our hypothesis is that the levels of risk imparted by age, presence of diabetes and diverse associated CVD are not the same. For that reason, we sought to determine the clinical predictors more closely related to CAD in 301 renal transplant candidates treated by hemodialysis [30]. CAD (≥70% stenosis) was found in 45% of cases, and the clinical variables significantly associated with CAD were diabetes, peripheral vascular disease and previous myocardial infarction. More importantly, the prevalence of CAD increased with the number of clinical predictors from 26% (none) to 100% (all present), whereas the incidence of events increased two-, four- and sixfold in those with diabetes, vasculopathy or previous myocardial infarction, respectively (*P* < 0.0001). Using these clinical parameters to select patients for invasive testing would allow the reduction of the prevalence of unnecessary angiography from 55% (when all patients undergo angiography) to 26%. However, missing the diagnosis of CAD in one-fourth of patients is still not satisfactory. Therefore, we are now seeking to refine this score by finding the precise influence of each relevant factor on prognosis. The preliminary results of this project have recently been reported [31]. In this way, we hope to find a means by which to reduce the number of invasive tests without compromising the ability to correctly identify patients with significant CAD. Meanwhile, we advocate coronary angiography for symptomatic patients, those with altered myocardial scans (either transient or fixed defects) and individuals with associated CVD, irrespective of symptoms or the results of noninvasive testing. Patients with diabetes types 1 and 2 who do not have any of the aforementioned characteristics, and regardless of how long they have had diabetes, do not undergo routine invasive evaluation.

### Management of coronary artery disease in patients with end-stage renal disease

#### *Clinical management*

In the current era of so-called “evidence-based medicine,” the treatment of patients with CKD and concomitant CAD should be based on solid data gathered from randomized clinical trials that included a large number of patients. Despite the indisputable fact that CAD is of great importance as a major determinant of cardiovascular morbidity and mortality in patients with CKD, our knowledge of how best to treat CAD in this special group of patients is less clear by far than what we already know regarding the management of CAD in patients without CKD. The main reason is that patients with CKD are consistently more often excluded from cardiovascular trials than patients with other comorbidities, such as diabetes, hypertension or smoking. In a paper by Charytan and Kuntz, who reviewed 86 cardiovascular trials that randomized more than 400,000 patients, 80% of the trials excluded subjects with end-stage renal disease (ESRD), whereas baseline renal function was reported in only 7% of the trials [32].

The simple transposition of a proven therapeutic strategy in reducing cardiovascular morbidity and mortality in patients with CAD and preserving renal function in those with CAD and CKD may not be so simple after all. Two recent clinical trials have proven that point exactly. Since the first publication of the Scandinavian Simvastatin Survival Study in the mid-1990s, statin therapy has become one of the cornerstones of the management of patients with proven CAD (and patients at high risk for CAD) and no CKD [33]. On the other hand, in both the 4D and AURORA studies, the use of atorvastatin or rosuvastatin, respectively, in patients at high cardiovascular risk undergoing hemodialysis failed to decrease the composite primary end point of cardiovascular death, nonfatal myocardial infarction or nonfatal stroke, even in those subgroups of patients with diabetes, a history of CVD or high levels of low-density lipoprotein (LDL) cholesterol or high-sensitivity C-reactive protein [34,35].

We must point out, however, that in both studies less than 40% of enrolled patients had any form of atherosclerotic CVD, including CAD, so those studies were not performed exclusively in patients with CAD and CKD. It is our understanding that if a patient presents with documented CAD, regardless of renal function status, statin therapy should be initiated and maintained, targeted to a level of LDL cholesterol below 70 mg/dl. In fact, two *post hoc* studies of the 4D and AURORA data indicate that statins may reduce cardiac events in selected groups of patients treated by dialysis [36,37]. In patients with a wide range of renal insufficiency, not necessarily on dialysis, the recent SHARP trial also showed a beneficial effect of simvastatin plus ezetimibe on the incidence of major atherosclerotic events [38]. There is a clear tendency toward recommending statin therapy according to the criteria for the general population in patients with CKD. On the other hand, it is still unclear if statins should also be recommended for CKD patients with no risk factors for coronary events as defined for the general population.

In light of the lack of studies specifically conducted in patients with CKD and CAD, we recommend following the current guidelines for the overall medical management of patients with chronic CAD proposed by the American Society of Cardiology and American Heart Association or the European Society of Cardiology, which have been advocated by the National Kidney Foundation Task Force on Cardiovascular Disease since the late 1990s [39]‐[41]. This multifaceted approach to overall cardiovascular risk reduction includes, in addition to lifestyle modifications (diet, physical activity and smoking cessation), statins and aspirin for all patients. β-blockers should be used in patients with symptomatic angina and/or after myocardial infarction as well as in patients with CAD and left ventricular dysfunction. Angiotensin-converting enzyme (ACE) inhibitors (or angiotensin type II receptor blockers (ARBs)) should be used in hypertensive patients with CAD with or without diabetes, as well as in patients with left ventricular dysfunction. Attention should be paid not only to initiating those drugs in patients with CAD and CKD on dialysis but also to keeping them on those drugs in cases of patients who undergo kidney transplantation, thereby minimizing the risk of a periprocedural cardiovascular event that could jeopardize the overall benefit conferred by an otherwise successful transplant. The possibility that renin-angiotensin blockers may cause serum creatinine levels to fall more slowly in recipients of live donor renal transplants still needs confirmation [42]. The dire consequences of coronary events during and in the early posttransplantation period should be always considered, however, even if some adverse side effects are anticipated.

This cardioprotective selection of drugs is increasingly being used in patients with CAD, however, for reasons that are still unclear, the prescription of these cardioprotective medications is less frequent among patients with CKD compared to the general population. In a previous study, we showed that in 119 patients with ESRD and CAD followed in a single center, the baseline use of aspirin and statins, in the range of 52% and 17%, respectively, was unexpectedly low [43]. In the same study, the use of ACE inhibitors (or ARBs) in 103 patients with diabetes and CKD was only 34%.

Thus, regarding the medical management of patients with CAD and stage V CKD, clinicians face two major challenges: (1) the lack of clinical trials specifically designed to assess the extension of the benefit of modern medical treatment and (2) the therapeutic nihilism that keeps physicians and healthcare providers from prescribing cardioprotective drugs with proven benefit in reducing cardiovascular mortality in the overall population.

#### *Myyocardial revascularization: percutaneous coronary intervention or coronary artery bypass graft*

The American Heart Association and American College of Cardiology recently jointly issued a document regarding criteria for the appropriateness of myocardial revascularization in patients with stable angina [44]. Briefly, myocardial revascularization procedures are indicated on the basis of three distinct elements: clinical presentation (that is, angina functional class), the results of noninvasive testing (stress-induced myocardial ischemia) and the extension of obstructive lesions. Patients who are more symptomatic and receiving optimal medical therapy with high-risk results evidenced by noninvasive tests and more extensive CAD should be referred for myocardial revascularization procedures. Again, in light of the lack of trials specifically designed to study patients with CAD and CKD, we are compelled to apply the same criteria established for patients with preserved renal function to patients with CKD.

There are two major caveats to that approach. The first one is that patients with ESRD are usually self-limited regarding physical activity, which may mask exercise-induced ischemia as a diagnostic clue to the severity of CAD. Moreover, even when patients do present with acute coronary syndrome, fewer with renal failure will have chest pain compared to those with normal renal function, making the clinical suspicion of CAD even more challenging [45]. The second issue relates to noninvasive testing for the diagnosis of CAD in patients with CKD. It is well-accepted now that the overall sensitivity and specificity for the diagnosis of CAD in patients with CKD are lower than those found in patients with normal renal function [46]. Therefore, clinicians may miss two of the three important elements that could lead to a clear indication for myocardial revascularization.

If one finally manages to overcome the previously alluded difficulties in the decision-making process and decides to refer a patient for a myocardial revascularization procedure, another question immediately follows: What kind of revascularization technique should be used, percutaneous or surgical?

As a general rule, the results of coronary interventions in patients with ESRD undergoing dialysis are worse than those performed in the general patient population. In various retrospective studies, perioperative death during coronary bypass graft (CABG) surgery in patients with ESRD undergoing dialysis varies from 5% to 20%, roughly three to four times higher than the rate in the general patient population. The 5-year mortality in CABG patients with ESRD who are undergoing dialysis is about 48%, compared with 15% in the general patient population [47].

Given the higher mortality rates in patients with CKD who undergo CABG surgery, referring a patient for such a risky procedure can be made only if the procedure not only provides symptom relief but also yields a clear reduction in mortality compared to those patients kept on medical treatment. In this regard, an early retrospective investigation showed that dialysis patients who underwent CABG surgery had a better prognosis than those treated medically [48]. A subsequent small, prospective study in hemodialysis patients with diabetes also showed that coronary intervention (surgery or angioplasty) was associated with reduced cardiac mortality and events [49]. It should be mentioned, however, that in both studies medical therapy was suboptimal by current standards. More recently, we looked at the impact of modern medical treatment of CAD compared to myocardial revascularization on the long-term occurrence of events in a registry of 230 patients with CKD and documented significant CAD (≥70% stenosis). In that study, 184 patients were kept on medical treatment and 46 were referred for myocardial revascularization, although 16 of them refused the procedure [50]. The event-free survival rates at 12 and 48 months were 86% and 61%, respectively, for patients kept on medical treatment alone and 97% and 79%, respectively, for those who had any revascularization procedure. Among those who refused the procedure, however, the event-free survival rate at 48 months was only 26%. We concluded that medical therapy in selected patients promotes acceptable long-term event-free survival rates and that failure to intervene may lead to an adverse outcome when myocardial revascularization was clearly indicated on the basis of the current guidelines.

In another study, Herzog and colleagues collected data from the US Renal Data System to compare the long-term survival of 15,784 dialysis patients after percutaneous angioplasty, coronary stenting or CABG surgery [51]. The 2-year all-cause survival rate was 56.4 ± 1.4% (CABG surgery), 48.2 ± 1.5% (angioplasty) and 48.4 ± 2.0% (coronary stenting). There was a statistically significant difference between the groups that indicated superior results of surgery over the other types of treatment.

Current data support previous observations regarding the overall superior benefit of CABG surgery over percutaneous coronary intervention (PCI) with drug-eluting stents (DESs) in patients with CKD on hemodialysis. In a small, nonrandomized study, Sunagawa *et al*. compared the event-free survival rates in patients with CKD on hemodialysis who underwent either CABG surgery (*n* = 29) or PCI (*n* = 75) [52]. They were able to show that at 2-year follow-up, the cardiac death rate was 0% for the patients who had CABG procedures and 16% for PCI-treated patients. During the later follow-up period, there were six deaths in the CABG group and twenty-seven (including six sudden deaths) in the PCI group. These authors concluded that the use of DESs in this patient population carries a higher risk for sudden death which might be due to stent thrombosis.

As we have discussed, the currently available data gathered is based on either (1) registries of patients with CKD and significant CAD that look retrospectively at outcomes according to different therapeutic strategies or (2) *post hoc* analysis of subgroups of patients with CKD prospectively enrolled in cardiovascular trials. What we are in great need of is a randomized clinical trial that enrolls only patients with CKD and significant CAD in whom both strategies (medical and invasive treatments) are equally justifiable based on current guidelines. Such a study has been proposed [53] and would provide the best evidence for choosing the right therapeutic strategy for treating CAD in this high-risk group of patients.

## Conclusion

CAD is a common and important complication in patients with advanced CKD. Because patients with CKD are frequently excluded from cardiovascular trials, no clear strategies have been developed specifically for the detection and treatment of CAD in these patients. That is one of the reasons for the erratic and disappointing results reported in the diagnosis and treatment of CAD in this population. Clinicians are in great need of randomized clinical trials that enroll solely patients with CKD in whom diagnostic and treatment strategies are tested based on current guidelines. Such studies would provide the best evidence for choosing the right strategy to screen and treat CAD in this high-risk group of patients.

## Competing interests

The authors declare that they have no competing interest.

## Authors’ contributions

JJGL, LHWG and FJP conceived the work and wrote the manuscript. All authors read and approved the final manuscript.
